# Can the Pupillary Light Reflex and Pupillary Unrest Be Used as Biomarkers of Parkinson’s Disease? A Systematic Review and Meta-Analysis

**DOI:** 10.3390/diagnostics15091167

**Published:** 2025-05-03

**Authors:** Aleksander Dawidziuk, Emilia Butters, Daniel Josef Lindegger, Campbell Foubister, Hugo Chrost, Michal Wlodarski, John Grogan, Paulina A Rowicka, Fion Bremner, Sanjay G Manohar

**Affiliations:** 1 Buckinghamshire Healthcare NHS Trust, Aylesbury HP21 8AL, UK; 2 Solvemed Inc., 16192 Coastal Highway, Lewes, DE 19958, USA; 3 Jesus College, University of Cambridge, Cambridge CB5 8BL, UK; 4 School of Psychology, Trinity College Dublin, D02 PN40 Dublin, Ireland; 5 University College London Hospitals NHS Foundation Trust, London NW1 2PG, UK; f.bremner@nhs.net

**Keywords:** pupillary light reflex, pupillary unrest, biomarkers, Parkinson’s disease, neurodegenerative disease

## Abstract

**Background/Objectives:** The pathological changes preceding the onset of Parkinson’s disease (PD) commence several decades before motor symptoms manifest, offering a potential window for identifying objective biomarkers for early diagnosis and disease monitoring. Among the primary non-motor features of PD is autonomic dysfunction; however, its precise assessment remains challenging, limiting its viability as a reliable biomarker. Both the pupillary light reflex (PLR) and pupillary unrest are regulated by autonomic pathways suggesting their potential as objective non-invasive indicators of the PD prodromal phase. This review systematically evaluates studies that compare PLR and pupillary unrest in individuals with PD and healthy controls to determine their utility as potential biomarkers of the disease. **Methods:** A systematic search strategy was designed to identify studies reporting PLR and pupillary unrest findings in PD patients. Searches were conducted across three databases (MEDLINE, Embase PsycINFO), supplemented by cross-referencing relevant studies found on Google Scholar. The literature search was last updated on 7 December 2020. Pupillometric parameters that permitted statistical synthesis included maximum constriction velocity (VMax), constriction amplitude (CAmp), and constriction latency (CL). Pooled incidence and effect sizes were determined using a random-effects model with an inverse variance DerSimonian–Laird estimator. The I^2^ statistic was used to assess study heterogeneity. When meta-analysis was not feasible, a qualitative analysis was undertaken. **Results:** The initial search yielded 219 references. Following deduplication and exclusion of ineligible studies, 31 papers were selected for review. Pupillometric data from 11 studies were incorporated into the meta-analysis. Effect sizes for PD patients were significant for VMax −0.92, (*p* < 0.01), CAmp −0.58, (*p <* 0.05), and CL 0.46, (*p <* 0.05). Measures of pupillary unrest were elevated in PD patients compared to controls, but evidence was limited to two studies. **Conclusions:** Pupillary constriction in response to light is characterised by reduced speed and amplitude in PD, with effect sizes suggesting potential clinical applicability. However, evidence regarding baseline pupillary variability remains insufficient, underlining the necessity for further research. Pupillary metrics represent a promising avenue for early PD detection, though their clinical utility is constrained by methodological heterogeneity and variations in disease duration among studies.

## 1. Introduction

Parkinson’s disease (PD) is one of the most common and fastest growing neurodegenerative diseases, affecting around 1 to 2 people per 1000 and has a lifetime risk of 2% for males and 1.3% for females [1]. While the aetiology of PD is likely multifactorial, the protein α-synuclein is a central component to the pathogenesis of the disease. The mechanism by which α-synuclein causes toxicity and contributes to neuronal death is still not fully understood. Mitochondrial dysfunction is widely considered to play a major role in the underlying mechanisms contributing to neurodegeneration in PD [2]. Age is a key risk factor for PD [3]. PD is a progressive disorder manifesting with both motor and non-motor symptoms. The most common motor features of PD include bradykinesia, muscle rigidity, tremor, and festinating gait [4,5,6], whereas the main non-motor symptoms are rapid eye movement (REM) sleep behaviour disorder (where patients enact their dreams), autonomic dysfunction, cognitive decline, depression, and olfactory deficits [4,5,6]. Remarkably, these non-motor symptoms precede the onset of motor abnormalities by up to 10 years in the “prodromal phase” of PD [7]. These early mental and somatic features provide clues to the early pathogenesis but rarely lead to neurological referral. Currently, diagnosing PD relies heavily on history and physical examination [8], particularly to distinguish idiopathic PD from other causes of Parkinsonism. These include multiple system atrophy (MSA), progressive supranuclear palsy (PSP), corticobasal disease (CBD), and dementia with Lewy bodies (DLB) [8,9,10], as well as vascular Parkinsonism, drug-induced Parkinsonism, and a wide range of rarer disorders. Dopamine transporter single-photon emission computed tomography (DaT-SPECT) and magnetic resonance imaging (MRI) both play a role in differential diagnosis. However, these additional specific tests are expensive, time-consuming, require additional visits to the hospital, and are not always diagnostic. Developing new non-invasive cheap tests could add to this diagnostic toolkit and help accurately identify PD at an earlier stage [11,12,13].

Autonomic dysfunction is a key marker in distinguishing synucleinopathy (PD, MSA) from other disorders and is an early feature. Measuring autonomic function is complex and involves tilt table tests, metaiodobenzylguanidine (MIBG) scans, sudomotor tests, or anal sphincter needle electromyography [14,15,16,17,18]. Importantly, the pupil is also under autonomic control, but pupillometry has not been proposed in diagnosing PD.

PLR is the pupil’s response to a step change in the intensity of light reaching the retina [19]. It is rapid and involuntary and, consequently, measurements are fast and objective. Measurements of the PLR provide a valuable indication of autonomic nervous system function, and deviations from the PLR norm may have clinical significance [20]. PLR measurements can be aggregated into four distinct phases, namely response latency, maximum constriction, pupil escape, and recovery. Light on the retina produces an afferent volley of impulses in the intrinsically photosensitive retinal ganglion cells (ipRGCs); these project to the olivary pretectal nuclei in the dorsal midbrain, which in turn have excitatory projections to the Edinger Westphal nuclei, causing an efferent volley in the parasympathetic fibres that supply the iris sphincter muscle in both eyes [21]. Patients have not only autonomic but also retinal involvement [22]. This may involve ipRGCs, which respond to light with a different time course and which may change pupillary dynamics in a characteristic way [23].

**Figure 1:**
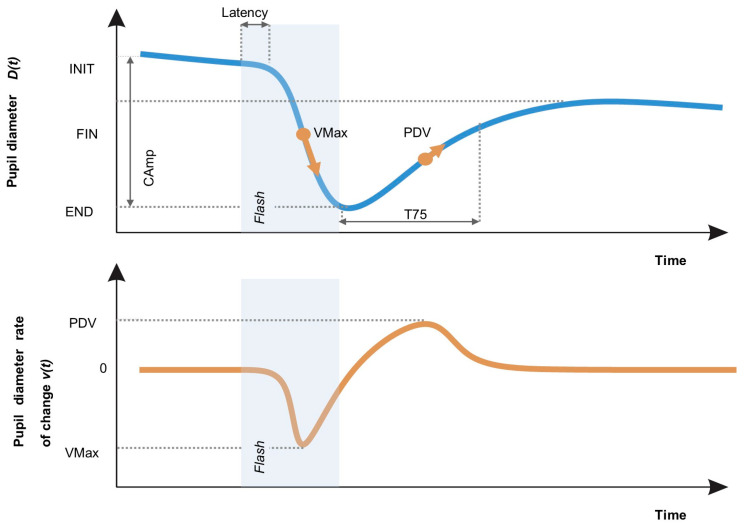
Representative schematic of the response to a flash stimulus. The top panel illustrates the modulation of the parameter pupil diameter following the flash, highlighting the MCV (maximum constriction velocity) in the constriction phase and PDF (peak dilation velocity) during subsequent recovery. CAmp (constriction amplitude) is indicated on the left side of the figure. The bottom panel depicts the pupil diameter rate of change v(t), characterised by an initial deviation followed by stabilisation. The shaded region represents the duration of the flash stimulus.

Response latency is defined as the interval between stimulus onset and response onset. In some cases, and particularly if the light stimulus is sustained for more than a second or two, after maximum constriction is achieved, the pupil may dilate a little (“escape”) [20]. Pupil dilation is either elicited by activation of the sympathetic nervous system (modulated by noradrenergic brain stem nuclei, the hypothalamus, and the central nucleus of the amygdala) or by inhibition of the parasympathetic outflow to the sphincter muscle (mediated by the reticular formation, locus coeruleus, and other cortical pathways) [24]. PLR parameters can be derived from each stage of the PLR via pupillometry (Figure 1). Heterogeneous experimental conditions and a lack of full reporting of methodologies in studies to date has led to some uncertainty regarding the usefulness of the PLR as a diagnostic tool. Figure 1 presents a schematic of a pupillogram and defines the commonly used pupillary light reflex outcome parameters.

The pupillary light reflex refers to the constriction of the pupil in response to light, followed by subsequent dilation, which occurs due to the opposing actions of the iris sphincter and dilator muscles. These muscles receive innervation from the parasympathetic and sympathetic divisions of the autonomic nervous system, respectively, allowing various PLR parameters to serve as markers of sympathetic or parasympathetic activity. As such, the PLR represents a valuable indicator of autonomic function and has been widely utilised in clinical practice. Dynamic pupillometry, a non-invasive and quantitative method for assessing the PLR, is now an established tool for evaluating traumatic brain injuries. Upon termination of the light stimulus, the initial phase of pupillary re-dilation is primarily driven by a reduction in parasympathetic activity, followed by a subsequent increase in sympathetic signalling to the iris dilator muscles.

Measurement of the PLR is a quick, low-cost, non-invasive method that is commonly used to measure a range of pathologies, such as optic and oculomotor nerve function, brainstem function, drug overdoses, and retro-orbital, neck, and cavernous sinus lesions. It has proven to be so reliable that it is one of the first screening tools used in the ambulance and emergency department and is performed hourly in neuro-critical patients to diagnose brain death. However, for other indications, heterogeneous experimental conditions and a lack of full reporting of methodologies in studies to date has led to mixed evidence regarding its effectiveness as a diagnostic tool. A variety of neurological, neurosurgical, and psychiatric conditions are known to alter pupillary light reflex parameters, including Parkinson’s disease [25,26,27,28,29,30], Alzheimer’s disease [25,29,31,32,33,34,35,36,37], schizophrenia [38,39], depression [31,40], and mild traumatic brain injury [41,42,43]. This suggests that there is great potential to expand its use in neurodiagnostics and disease management [20,44,45,46,47,48,49].

PLR is affected by a number of environmental factors such as the intensity, luminance, colour, and angle of light stimuli and background illumination [50], as well as external factors (including retina and optic nerve health, medication, psychological state including level of alertness, a task’s cognitive demand, and other non-visual, auditory and reward cues) [20,51,52]. For example, pupillary dilation in response to rewards (e.g., monetary incentivisation) has been shown to be blunted in PD [53], which may be a reflection of the clinical apathy associated with PD [54] or a predictor of impulsivity [55]. It may even measure prodromal features of PD [56]. Recent work has shown that PD may also affect intrinsically photosensitive retinal ganglion cells, which trigger different PLR response dynamics than rod and cones as a function of wavelength [50]. Thus, measuring PLR parameters at a variety of wavelengths could allow for the separation of controls from PD patients and even people with prodromal features [57,58]. This wavelength effect may also permit the discrimination of patients with retinitis pigmentosa or congenital amaurosis who have attenuated PLR to blue light [59].

This review aims to systematically evaluate the utility of PLR parameters and pupillary unrest as quantitative biomarkers of Parkinson’s disease diagnosis. Moreover, we aim to meta-analyse the existing evidence to confer a more robust assessment of the value of each of these parameters in the diagnostic process. Meta-analysis will help partition the effects of Parkinson’s disease using key covariates including age, disease severity (Unified Parkinson’s Disease Rating Scale (UPDRS) part III score, Hoehn and Yahr (H&Y) stage), disease duration, medication status, and cognitive function (Mini-Mental State Examination (MMSE) score). The previous literature reviews have been either narrative reviews focused on single measures or were published many years ago—and a considerable number of original research articles have been published since [60,61]. Furthermore, we have not found any previous meta-analyses of how Parkinson’s disease impacts the ocular measures explored in this work.

## 2. Materials and Methods

### 2.1. Search Strategy

This systematic review was conducted with adherence to the Preferred Reporting Items for Systematic Reviews and Meta-Analyses (PRISMA) guidelines [62]. An electronic search of Embase (1947 to December 2020), MEDLINE (1946 to December 2020), and PsycINFO (1806 to December 2020) was conducted with the following combinations of terms: (“Parkinson’s” OR “Parkinson’s Disease” OR “Parkinson’s” OR “Parkinsons Disease” OR “Parkinson” OR “Parkinson Disease” OR “Parkinsonism” OR “Paralysis Agitans”) AND (“flash response” OR “pupillary reflex” OR “pupil reaction” OR “pupillary reactivity” OR “pupillary response” OR “pupil response” OR “pupillary unrest” OR “pupillary motility”) in the abstracts of the records. The search was restricted to studies conducted on human participants and published in English. Further relevant records were located via a Google Scholar search and by examining the reference lists of the included studies. The literature search was last updated on 7 December 2020.

### 2.2. Eligibility Criteria

#### 2.2.1. Inclusion Criteria

The publications were included in the review only if they met all the following criteria:

- Original experimental studies collecting data on human subjects.
- Studies investigating a cohort of patients diagnosed with Parkinson’s disease.
- Studies measuring PLR parameters or pupillary unrest.

#### 2.2.2. Exclusion Criteria

Publications non-experimental in nature (reviews, editorials, letters, commentaries, short surveys), conference abstracts, dissertations and methodological papers not involving and human subjects were excluded.

### 2.3. Data Extraction

Relevant studies were initially screened by two authors (AD, EB) based on their titles and abstracts using the Covidence platform (Veritas Health Innovation Ltd., Melbourne, VIC, Australia). Any discrepancies in the selection process were resolved through discussion with the senior author (PR). Full-text articles that met the inclusion criteria were retrieved and assessed for eligibility. A summary of the studies included in the final review is presented in Table 1. Data extraction from the selected studies was conducted using Microsoft Excel for Mac Version 16.42 (Microsoft Corporation, Redmond, WA, USA). The following study details were documented: author, study design, sample size, inclusion and exclusion criteria, duration since Parkinson’s disease diagnosis, disease stage, presence of cognitive symptoms, medication status, ocular assessment methods, study protocol, and measurement outcomes.

### 2.4. Quality and Risk of Bias Assessment

To ensure appropriate assessment of the selected publications, quality and risk of bias were assessed by two independent assessors (AD, EB). Quality of the studies included in the review was assessed using the Newcastle–Ottawa Scale [63]. Publication bias was assessed graphically using a Funnel plot and quantitatively using the Begg rank correlation test [64] and Egger regression asymmetry test [65]. If publication bias was present, denoted by a *p* < 0.05 in these latter tests, Duval and Tweedie’s trim and fill test was used to adjust the data for such bias [66].

### 2.5. Data Analysis

Data were extracted as mean values with standard deviations. These data were then converted to a standardised mean difference (SMD) between PD patients and controls, with corresponding 95% confidence intervals (CIs), and studies were weighted using the generic inverse variance method. Heterogeneity between studies was assessed using Cochrane Q and I^2^ statistics [67]. An I^2^ value of over 50%, with *p* < 0.05, was considered to signify significant heterogeneity. The overall effect was calculated using a random-effects model with the DerSimonian–Laird estimator [68]. A meta-regression with restricted maximum likelihood estimation was carried out to compute the effect of covariates, which may influence between-study heterogeneity. If this meta-regression yielded positive results, a permutation test was run to verify these results [69]. These covariates included age, PD severity, PD duration, Hoehn and Yahr stage, medication status, Mini-Mental State Examination score, and Unified Parkinson’s Disease Rating Scale part III score. A leave-one-out sensitivity analysis was also performed by iteratively removing each study and re-estimating the pooled effect size to assess whether the overall effect size was significantly influenced by a single study [70]. In this approach, we systematically removed one study at a time from the meta-analysis and recalculated the pooled effect estimate. This allowed us to assess whether any single study had a disproportionate influence on the overall results. The meta-analysis was carried out using R Studio Version 2024.12.1 [71].

## 3. Results

### 3.1. Study Selection

**Figure 2:**
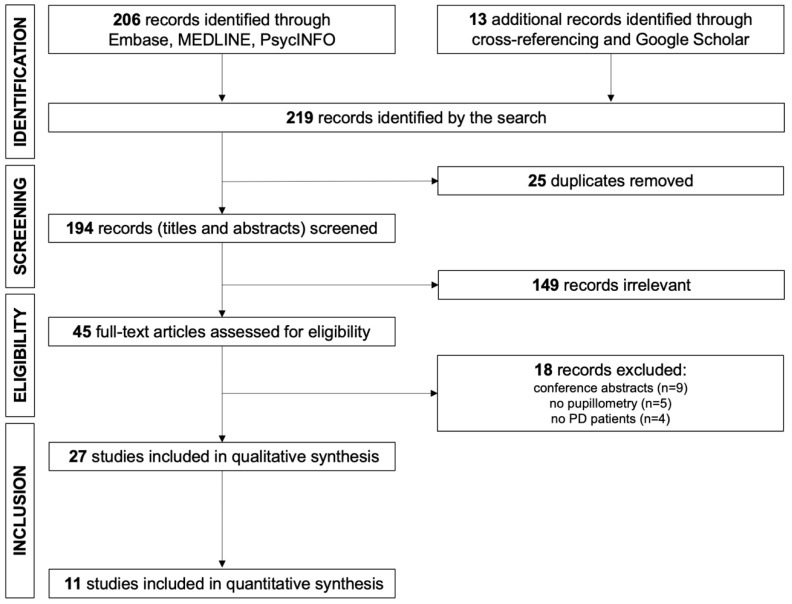
PRISMA flow diagram detailing exclusions throughout each stage of study selection (PD = Parkinson’s disease) [62].

Figure 2 displays the study selection process. Initially, the search yielded 219 records, which was reduced to 194 after deduplication. Following title and abstract screening and initial exclusions, 45 full-text articles were assessed for eligibility. Of these 45 articles, 11 studies contained pupillary data and were thus included for quantitative analysis, with a further 16 studies included for systematic review. Characteristics of the studies included in the review as well as their main findings are summarised in Table 1.

### 3.2. Assessment of Quality

**Figure 3:**
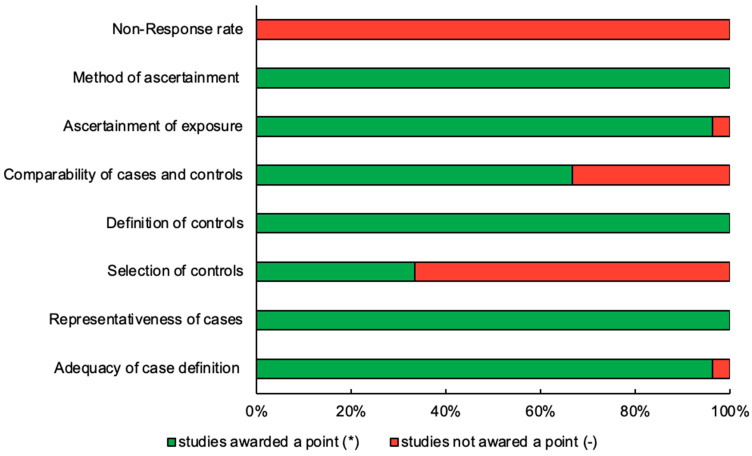
Newcastle–Ottawa Scale assessment of the studies included in the review. The percentage of studies awarded a point in each category is represented by an asterisk (*), while dash (-) represents studies not awarded a point.

Quality was formally assessed using the Newcastle–Ottawa Assessment Scale for case–control studies, which revealed moderate variability in the quality of the included papers. In total, 18 papers were rated as good quality and nine as poor-quality based on the Agency for Healthcare Research and Quality (AHRQ) standard (Table 2, Figure 3).

### 3.3. Evaluation for Publication Bias

**Figure 4:**
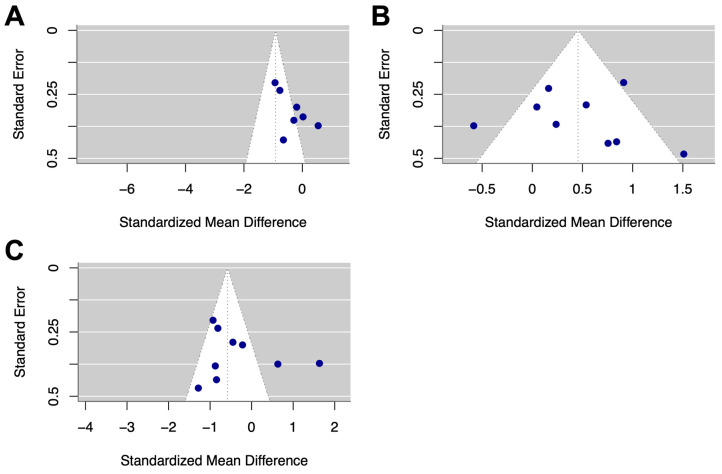
Funnel plots of studies for (**A**) VMax, (**B**) CL, and (**C**) CAmp. Standardised mean difference from each study is shown on the x axis and standard error is shown on the y axis.

A visual inspection of funnel plots for CL and CAmp revealed little asymmetry for these metrics (Figure 4). No significant publication bias was identified through Egger’s regression-based test [64] for VMax (*p* = 0.216), CL (*p* = 0.984), and CAmp (*p* = 0.892) or through Begg’s rank correlation test [65] for VMax (*p* = 0.612), CL (*p* = 0.761), and CAmp (*p* = 0.727) [64,65].

### 3.4. Pupillary Light Reflex in Parkinson’s Disease

#### 3.4.1. Maximum Contrition Velocity (VMax)

**Figure 5:**
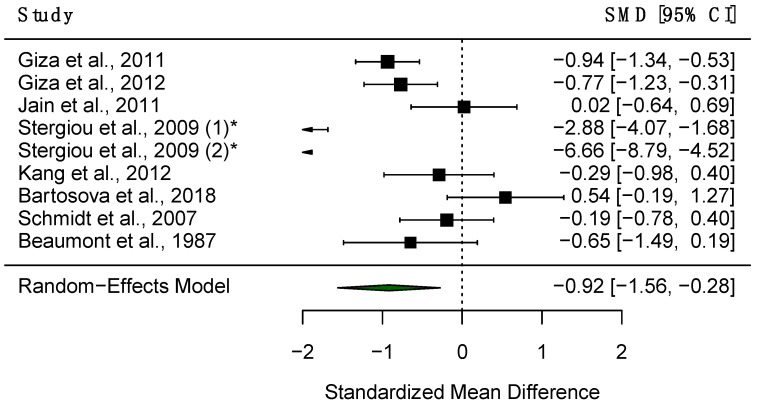
Forest plot demonstrating differences in VMax between PD patients and HC. The black boxes represent the SMD for each trial and the arms represent the 95% CI. The size of these boxes represents the relative weight of each study. The green diamond represents the overall SMD of the trials. * Study included twice, as two separate PD populations were tested.

**Figure 6:**
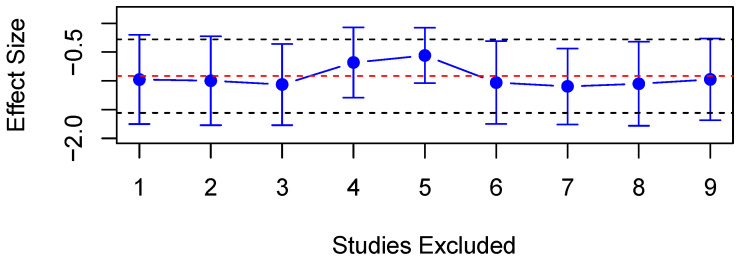
Sensitivity analysis of all the included studies for VMax. Overall effect size shown as red dashed line with 95% CI shown as black dashed line.

Ten studies assessed VMax in both PD patients and HC; of these, eight studies had data available for meta-analysis. A random-effects meta-analysis found significantly decreased VMax in PD patients compared to HC (SMD = −0.92, 95% CI = −1.56 to −0.28, *p* < 0.01, Figure 5). Additionally, high heterogeneity was observed amongst studies reporting VMax in PD patients and HC (*Q* = 65.45, *df* = 8, *p* < 0.001, I^2^ = 87.39%). A meta-regression was thus carried out to explore the source of such heterogeneity. The proportion of male participants (adjusted R^2^ = 65.4%, *p* < 0.001) and mean age (adjusted R^2^ = 85.8%, *p* < 0.001) were significant predictors, with one study finding a significant correlation between VMax and patient age [72]. A sensitivity analysis revealed that no single study influenced the results significantly (Figure 6). Some evidence for differences in VMax shows it to be lower in patients compared to controls [28,74], but four studies observed no significant differences [73,79,80,81].

#### 3.4.2. Constriction Latency (CL)

**Figure 7:**
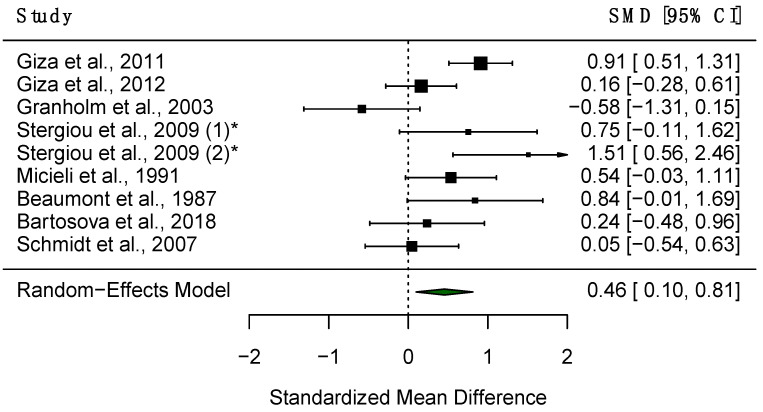
Forest plot demonstrating differences in CL between PD patients and HC. The black boxes represent the SMD for each trial and the arms represent the 95% CI. The size of these boxes represents the relative weight of each study. The green diamond represents the overall SMD of the trials. * Study included twice, as two separate PD populations were tested.

**Figure 8:**
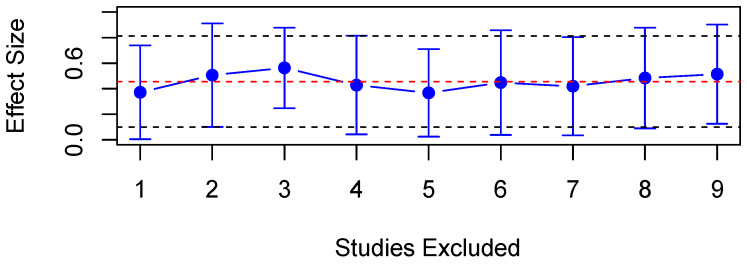
Sensitivity analysis of included studies for CL. Overall effect size shown as red dashed line with 95% CI shown as black dashed line.

Eleven studies reported results for CL in both PD patients and healthy controls, of which eight were provided data such that they could be assessed quantitatively. A random-effects meta-analysis found significantly prolonged CL in PD patients compared to HC (SMD = 0.46, 95% CI = 0.10 to 0.81, *p* < 0.05, Figure 7), with substantial heterogeneity observed across studies (Q = 22.69, df = 8, *p* < 0.01, I^2^ = 64.75%). A meta-regression was carried out across studies to explore the source of such heterogeneity, and the mean age of participants was found not to be a significant predictor with *p* > 0.05. A sensitivity analysis also revealed that no single study influenced the results significantly (Figure 8). Four studies found significantly greater CL in patients [26,27,30,72]; however, five studies found no significant difference between PD patients [28,29,73,80,81].

#### 3.4.3. Constriction Amplitude

**Figure 9:**
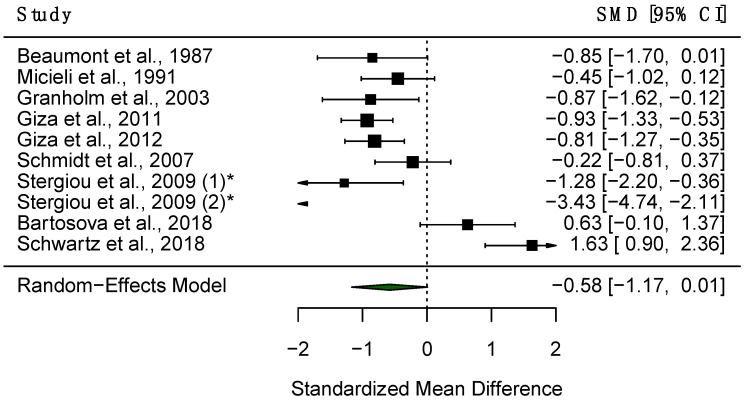
Forest plot demonstrating differences in CAmp between PD patients and HC. The black boxes represent the SMD for each trial and the arms represent the 95% CI. The size of these boxes represents the relative weight of each study. The green diamond represents the overall SMD of the trials. * Study included twice, as two separate PD populations were tested.

**Figure 10:**
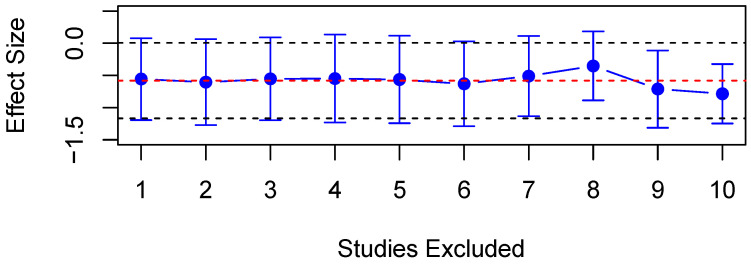
Sensitivity analysis of included studies for CAmp. Overall effect size shown as red dashed line with 95% CI shown as black dashed line.

Twelve studies explored CAmp in PD patients and HC, of which nine studies reported data which were thus included in the quantitative analysis. The random-effects meta-analysis found significantly increased CAmp in PD patients compared to HC (SMD = −0.58, 95% CI = −1.17 to 0.01, *p* < 0.05, Figure 9), and substantial heterogeneity was observed within this analysis (*Q* = 72.38, *df* = 9, *p* < 0.001, I^2^ = 87.57%). A meta-regression found that the mean age of participants was a significant predictor of heterogeneity (adjusted R^2^ = 15.3%, *p* < 0.05), with one study finding a significant correlation between CAmp and the mean PD patient age [72]. A sensitivity analysis also revealed that no single study influenced the results significantly (Figure 10). Six studies found CAmp to be significantly lower in patients compared to controls [26,27,29,30,72,74], and the pre-stimulus diameter of the pupil has been observed to be significantly smaller in patients [24,85]. However, three studies found no significant differences between PD patients and controls [21,73,81], alongside no influence of medication on CAmp [53]. Additionally, one study found no effect of the group between PD patients and controls on pupillary responses to emotional film clips [83].

Studies exploring other metrics of the PLR did not contain enough data to perform a quantitative analysis and were therefore not systematically evaluated. Five studies investigated minimum pupil radius (RMin) and minimum pupil diameter. Four studies [26,27,28,72] showed that there were no significant differences for PD patients and HCs; one study showed that the mean minimum pupil diameter was higher in PD patients under L-DOPA [81]. Alternatively, the maximum pupil diameter has been observed to be significantly greater in PD patients compared to HC [81]; however, two studies found no significant difference [72,85]. The maximum acceleration of constriction (AMax) has also been found to be significantly slower in patients compared to controls in four studies [26,27,28,74]. There is also mixed evidence for time to maximum miosis (TMiosis), with one study finding no differences between patients and controls [28] and two studies with significantly longer TMiosis in patients compared to HC [21,30].

A number of studies measured baseline pupil size in both PD patients and controls. Ten studies found no differences in the initial radius (IR) between patients and controls [24,26,27,28,29,54,76,77,84,85], and one study did not test for significance [83]. However, two studies found a significantly larger pupil diameter in PD patients compared to controls [21,53] whereas one study found pupillary area to be significantly reduced in younger PD patients compared to age-matched HC, with no difference in older patients [74]. A significantly greater pupillary diameter has also been observed in patients on medication compared to those off medication [53,54,55], in line with α-adrenergic effects of levodopa metabolites [88].

Pupil dilation can also be used to assess cognitive effort. The evidence for differences in cognitive pupillary dilation between PD patients and controls is mixed. One study found no differences in a letter–number sequencing test [82]; however, one study found significant differences in the left but not the right eye during a verbal reasoning task [87] and another found differences in a dual- and single-task balance task [86]. Cognitive effort may operate via similar autonomic pathways as motivation, which also causes pupillary dilation [53]. Motivation is also critically dependent on dopamine and is reduced in PD patients OFF medication. In line with this, the pupillary motivation response is larger in patients ON dopaminergic medication compared to OFF alongside the drug effect on baseline pupil size [54,55].

Various studies suggest the presence of within-group differences across pupillary metrics. CAmp has been found to be significantly lower in PD patients with cognitive impairment (CI) compared to those without CI (N-CI) and controls [28]. Additionally, task-evoked pupillary response, a measure of cognitive workload, is significantly greater in PD patients with CI compared to N-CI PD patients and controls [52]. Significantly lower VMax and Amax have also been identified in PD patients with CI compared to N-CI patients [28]. In patients with prodromal features, CI increases the speed of re-dilation (sometimes termed the “post-illumination pupillary response”). The influence of PD severity and duration of disease also could not be quantitatively assessed due to the lack of covariate data in the included studies. However, one study proposed that as PD severity increases, patients transition from having normal pupils to a physiologically dilated state, then to a hyper-cholinergic state (PLR more similar to having pilocarpine drops), and finally to a low-cholinergic state (PLR more similar to having tropicamide drops) [74].

Multiple studies have explored the effect of parasympathomimetic and sympathomimetic compounds on the PLR. Significant differences have been observed in both pupillary change and pupillary sensitivity between PD patients and HC in response to varying concentrations of pilocarpine hydrochloride (PL) and dipivefrine hydrochloride (DPE) [76], in which a greater change was observed for PD patients with longer disease durations [77] alongside visual disturbances such as blurred vision and involuntary eyelid closure [76]. Significant pupillary change has also been observed in MSA patients only in response to DPE [77], and a further study found significant differences between MSA and both PD patients and HC in mean pupil diameter [75]. These findings suggest a potential for differential diagnosis between neurodegenerative disorders using pupillary metrics.

Re-dilation of the pupil after the PLR often shows no difference in patients with PD [30,72,73,80], although one study found re-dilation velocity to be slower in PD [74].

### 3.5. Pupillary Unrest

Two papers explored differences in pupillary unrest between PD patients and controls, in which significantly greater pupillary unrest in patients was observed in two studies, as measured by the Pupillary Sleepiness Test [78,79]. Despite the limited data available for this metric in PD, pupillary unrest is positively associated with PD severity, as assessed by the UPDRS part III scale [78]. Alongside a lack of influence of PD medication on pupillary unrest [79], these studies support the potential for using this measure as a biomarker of PD; however, further larger scale studies are required to explore this possibility.

## 4. Discussion

Here, we meta-analysed 11 studies comparing quantitative pupil measures in PD against controls. Despite the methodological quality of the included papers being variable, we found evidence for differences in the three most-commonly measured pupil parameters. There was evidence for longer latency, slower peak velocity, and larger amplitude of the constriction to light. Studies showed heterogeneous results, and not enough evidence was available to ask what other variables drove this heterogeneity. There were too few studies of pupillary unrest to draw quantitative conclusions.

### 4.1. PLR Parameters and Pupillary Unrest as PD Biomarkers

There is a close biological link between pupil responses and physiology [89]. If the variables that alter PLR can be controlled, such as illumination, arousal, time of day, and convergence, then PLR could provide a direct and objective readout of brainstem neurodegeneration. By probing the neural system using a pulse of light, we obtain a dynamic response which provides more robust information than the initial diameter. Moreover, the shape of the response may carry much richer information about cholinergic, adrenergic, cognitive, and circuit-level properties of brain function. A potentially exciting direction for future work is therefore to probe the light response using more complex input patterns [90,91,92].

#### 4.1.1. Findings of the Qualitative Synthesis

Autonomic functions are notoriously difficult to test in clinic settings. Various tests used in clinical diagnostics either have practical limitations or cannot be well standardised. Thus, a more precise investigation of quantitative pupillometry could provide a much more accessible objective test that could be useful in PD, MSA, diabetic neuropathy, amyloidosis, Guillain–Barre syndrome, and primary autonomic failure, amongst others.

In general, effect sizes were small. This could be due to variability between patients in lighting, time of day, attention, or arousal. However, most of the reviewed studies demonstrated that PD was characterised by changes in multiple pupillary parameters together. This naturally suggests that combining parameters together may offer greater sensitivity and specificity than any single parameter alone. Furthermore, the studies reviewed qualitatively suggest that other subtle features of the pupillary response, which are not captured by standard parameters, may provide additional signals with which to differentiate PD.

#### 4.1.2. Effect Size

An effect size of 0.92, as we obtained for VMax, is usually reported as “large” [93], enough to provide clinical utility. Depending on the distribution of the data, this could in principle permit the classification of PD vs. controls with up to 80% accuracy.

### 4.2. Limitations and Future Directions

Even though this meta-analytic review has managed to identify three PLR parameters with potential to be used as biomarkers of PD, there are several limitations of these findings. Firstly, there is significant variability in the methodologies of measuring PLR and reporting outcomes. Due to this barrier, only 11 articles which reported raw pupillometric data for both PD patients and healthy controls were included in the meta-analysis. For 16 papers, only qualitative appraisal was possible; the inclusion of numerical findings from these studies could potentially have improved the strength and reliability of the results presented. The varying ages, medications, co-morbidities, and lighting conditions all add to the heterogeneity. These factors might account for the differences in mean values of the same PLR parameters in different studies. Overall, the variability across studies limits our ability to identify normative ranges and cutoffs for diagnosis. Two possibilities would be to standardise testing conditions or to correct for the effects of other factors in analysis and interpretation.

Secondly, the PD patients recruited by the studies included in the review had long-established diagnoses. It remains an open question as to whether the differences hold at the time of diagnosis or in the prodromal phase. Most studies did not investigate subgroups stratified based on disease duration, severity of symptoms, or dementia status. As the reporting of these population characteristics used in the studies is heterogenous, the reliability of co-variate analysis remains limited. While documented REM sleep disorder in the study population indicates a possible presence of a neurodegenerative disease such as PD [94], a lack of stratification according to clinical disease severity limits correlation with pupillometric diagnostics [79]. The lack of correlation of biomarkers with disease severity could indicate that they are sensitive in all stages of PD, even in early disease. This is not unexpected, because PLR measures autonomic function, which is one of the earliest manifestations in PD, detectable 10–20 years before diagnosis [95].

REM sleep behaviour disorder is a syndrome in which patients enact their dreams, and it carries an 80% chance of conversion to PD over 10 years. Autonomic dysfunction (constipation, urinary, cardiovascular, and erectile) is common in REM sleep behavioural disorder (RBD) and may independently predict neurodegeneration [96,97] even more strongly than RBD itself [98]. Pupillometry is rarely assessed in prodromal diseases such as RBD and could offer a quick and sensitive index of autonomic dysfunction.

The patients in most studies were elderly, with severe symptoms reflecting an extensive loss of dopaminergic neurons. In these populations, the value of establishing a new biomarker is relatively low, because any potential disease-modifying treatments are more likely to be effective in earlier stages of PD progression.

To overcome these challenges in the future, new case–control studies with large sample sizes need to be conducted to validate proposed PLR parameters as qualitative biomarkers of PD. Community-collected data with a mobile application is capable of providing such data sets. The sample of PD patients involved in these investigations should be enriched for early PD and/or patients with prodromal features to allow for subgroup analysis and establishing which PLR parameters are most effective at each stage of PD progression. A deeper understanding of pupil physiology would help us to correct for variables that currently add noise to the measurement. Stronger mechanistic knowledge about the relationship between autonomic, retinal, and brainstem functions would provide the PLR with a strong theoretical footing and allow richer photo-perturbations and dynamic metrics to be developed, which can control for or cancel out confounding variables.

We identified differences in three parameters, but we cannot establish from these data how correlated they are with each other. Studies to date have considered each index independently, but if they are relatively uncorrelated, then multivariate approaches hold promise to increase utility further. If correlations are low, then integrating information from multiple metrics using machine learning could significantly increase discriminability. In fact, instead of estimating a few parameters, rich time-series data could be fed into a neural network that models the response. With sufficient data, this could partial out covariates to produce a probabilistic risk for PD diagnosis, either to aid specialists or provide screening.

## 5. Conclusions

Pupil metrics, especially the peak velocity, latency, and amplitude of pupillary constriction to light, offer a promising avenue for the detection of PD. To establish these parameters as quantitative biomarkers of PD, further large-scale case–control studies with consistent methodologies and reporting, as well as samples of patients in different stages of PD progression, are required.

## Figures and Tables

**Table 1:** Studies investigating pupillary light reflex parameters and pupillary unrest in Parkinson’s disease patients and healthy controls.

Reference	Population Characteristics							Study Characteristics			
	Number of Participants (PD/HC)	Mean Age (Years)	% Male	Mean Duration of Disease (Years)	Mean UPDRS III Score	Mean H&Y Score	Levodopa Equivalent Dose (mg/Day)	Pupillometric Device	Stimulus Used	Main Findings	Limitations
Beaumont et al., 1987 [72]	12/11	61.3	-	-	-	-	-	IR-sensitive TV camera	IR light	Longer CL and decreased CAmp in PD patients.	-
Orskov et al., 1987 [73]	23/10	62.4	-	6.5	-	2.7	-	IR-sensitive camera	Photostimulator	No difference in CL, VMax, and CAmp between PD patients and controls.	-
Sugiyama and Utsumi, 1989 [74]	24/30	64.0	-	4.70	-	2.4	-	IR video pupillography	IR light	Lower AMax, CAmp, and VMax in PD patients.	-
Micieli et al., 1991 [30]	23/26	56.2	71.4	2.3	-	1.8	-	IR binocular TV pupilometer	Photostimulator	Longer CL and greater CAmp, CT and greater light adaptation in PD patients.	-
Granholm et al., 2003 [29]	15/15	69.8	53.3	-	-	-	-	Binocular IR pupillograph	Photostimulator	Greater RMin and decreased CAmp in PD patients.	Small sample size; potential effects of medication.
Schmidt et al., 2007 [75]	19/27	62.0	60.9	3.50	-	2.0	488	Compact integrated pupillograph	Photostimulator	Lower CAmp, ID and CT in PD patients.	-
Hori et al., 2008 [76]	40/17	62.5	63.0	8.30	-	2.9	-	IR-sensitive camera	IR iriscorder	Pupillary sensitivity to PL and DPE greater in PD patients.	Did not assess the effect of corneal permeability.
Stergiou et al., 2009 [28]	22/11	72.7	45.5	5.10	-	-	-	Charged coupled device	IR light	Lower AMax and VMax in PD patients. Lower AMax, VMax, and CAmp in PD-CI compared to PD-NCI.	-
Yamashita et al., 2010 [77]	40/20	63.7	53.3	2.70	-	-	-	Binocular Iriscorder	-	Pupillary sensitivity to DPE and PL greater in PD patients.	-
Dietz et al., 2011 [24]	14/12	71.7	65.4	-	25.1	2.3	737.1	Eye tracker	IR light	Attenuated light reflex in PD patients.	Potential effects of medication.
Giza et al., 2011 [26]	66/44	64.2	58.2	4.7	21.2	2.0	-	Charge-coupled high-speed digital camera	Photostimulator	Longer CL and decreased CAmp, VMax, and AMax in PD patients.	-
Jain et al., 2011 ^a^ [78]	14/17	61.7	58.1	-	10.1	1.7	-	IR video pupillography	IR light	Pupillary unrest greater in PD patients.	Small sample size; limited range of PD severity; limited range of arousal symptoms; cross-sectional design.
Jain et al., 2011 ^b^ [79]	17/18	62.5	62.9	-	11.1	1.7	516	IR video pupillography	IR light	Pupillary unrest greater in PD patients. No difference in VMax.	Small sample size; limited range of PD severity; residual effects of medication (withheld for 12 h before testing).
Giza et al., 2012 [27]	35/44	63.7	58.2	-	-	-	-	Charge-coupled high-speed digital camera	Photostimulator	Longer CL and decreased CAmp, VMax, and AMax in PD patients.	-
Kang et al., 2012 [80]	15/18	62.8	69.7	-	10.7	1.7	516	-	Photostimulator	No difference in VMax between PD patients and controls.	Small sample size; cross-sectional design; limited range in PD severity; possibility of residual effects of longer-acting anti-Parkinsonian medications affecting results after withholding medications for 12 h.
Manohar and Husain 2015 [53]	16/22	63.8	46.3	-	23.1	1.8	-	Eye tracker	Photostimulator	Greater ID in PD patients. No difference in CAmp.	-
Muhammed et al., 2016 [54]	40/20	52.9	42.9	5.0	19.4	1.3	-	Eye tracker	Auditory cue	No difference in ID and reward sensitivity between PD patients and controls.	-
Wang et al., 2016 [21]	22/19	68	56.1	6.10	29.5	2.4	685	Eye tracker	Visual stimulus	TMiosis longer and ID greater and dilation size smaller in PD patients.	-
Ranchet et al., 2017 [52]	21/16	67	64	5.2	27.6	2.0	300 (CI), 463 (NCI)	Eye tracker	Prosaccade & antisaccade tasks	Greater cognitive workload for PD-CI in pro-saccade task and for PD-NCI in anti-saccade task.	Potential effects of medication; large heterogeneity in CI group; task design.
Bartosova et al., 2018 [81]	16/14	58.2	70.0	10.0	-	-	532	Monocular IR pupillograph	Photostimulator	Higher RMin and RMax in PD patients, no difference in VMax and CL.	-
Kahya et al., 2018 [82]	16/10	-	46.2	6.0	30.0	2.3	750	Eye tracker	-	No difference in cognitive load between PD patients and controls.	Small sample size; groups not matched for sex.
Schwartz et al., 2018 [83]	17/22	63.6	51.3	9.90	-	-	-	Eye tracker	Emotional video stimuli	No differenced in pupil dilation and diameter between PD patients and controls.	Unable to fully control the brightness and contrast between video clips.
Srivastava et al., 2018 [84]	12/10	36.1	100.0	5.2	-	-	-	Eye tracker	Visual stimulus	Reduced pupil width in PD patients.	Small sample size; influence of medication not taken into account.
Araga et al., 2019 [85]	45/20	64.3	50.8	-	28.9	2.9	644.3	Eye tracker	IR light	Reduced RMin in PD patients, no difference in ID.	Did not evaluate retinal function; did not compare any other diseases with Parkinsonism syndrome.
Drew et al., 2020 [55]	26/31	-	-	4.9	22.9	1.3	497.15	Eye tracker	Visual targets	ID greater in PD patients ON compared to OFF medication.	Patient groups might not be representative of typical cases; sample sizes relatively small; potentially heterogeneous; baseline factors that might also have been potential confounding factors.
Kahya et al., 2020 [86]	33/35	69.0	48.5	-	44.0	2.3	302.8	IR camera and eye tracker	-	Greater ICA in PD patients with which correlates with pupillary response in PD patients.	Fluctuations in cognitive and motor performance in PD; relies on self-report.
Moon et al., 2020 [87]	19/10	66.2	58.6	5.70	30.9	2.1	749.3	Eye tracker	Cognitive tasks	Greater cognitive load in PD patients.	Small sample size; potential effects of medication; unmatched sex distribution; unequal participant numbers; cross-sectional design.

^a^, Movement disorders; ^b^, Parkinsonism-related disorders.

**Table 2:** Newcastle–Ottawa Scale assessment of the studies included in the review, with respective quality scores based on the Agency for Healthcare Research and Quality (AHRQ) standard.

Reference	Selection				Comparability	Exposure			Quality Score (AHRQ Standard)
	Adequacy of Case Definition	Representativeness of Cases	Selection of Controls	Definition of Controls	Comparability of Cases and Controls	Ascertainment of Exposure	Method of Ascertainment	Non-Response Rate	
Beaumont et al., 1987 [72]	*	*	-	*	-	* -	*	-	Poor
Orskov et al., 1987 [73]	*	*	-	*	** (age, sex)	* -	*	-	Good
Sugiyama and Utsumi, 1989 [74]	*	*	-	*	** (age, sex)	* -	*	-	Good
Micieli et al., 1991 [30]	-	*	-	*	-	- -	*	-	Poor
Granholm et al., 2003 [29]	*	*	-	*	-	* -	*	-	Poor
Schmidt et al., 2007 [75]	*	*	-	*	** (age, sex)	* -	*	-	Good
Hori et al., 2008 [76]	*	*	-	*	** (age, sex)	* -	*	-	Good
Stergiou et al., 2009 [28]	*	*	*	*	** (age, sex)	* -	*	-	Good
Yamashita et al., 2010 [77]	*	*	-	*	-	* -	*	-	Poor
Dietz et al., 2011 [24]	*	*	*	*	-	* *	*	-	Poor
Giza et al., 2011 [26]	*	*	-	*	* (age)	* -	*	-	Good
Jain et al., 2011 a [78]	*	*	-	*	** (age, sex)	* *	*	-	Good
Jain et al., 2011 b [79]	*	*	-	*	** (age, sex)	* *	*	-	Good
Giza et al., 2012 [27]	*	*	-	*	* (age)	* -	*	-	Good
Kang et al., 2012 [80]	*	*	-	*	** (age, sex)	* -	*	-	Good
Manohar and Hussain 2015 [53]	*	*	-	*	* (age)	* -	*	-	Good
Muhammed et al., 2016 [54]	*	*	*	*	* (age)	* -	*	-	Good
Wang et al., 2016 [21]	*	*	*	*	* (age)	* -	*	-	Good
Ranchet et al., 2017 [52]	*	*	*	*	-*(sex)	* -	*	-	Poor
Bartosova et al., 2018 [81]	*	*	-	*	-	* -	*	-	Poor
Kahya et al., 2018 [82]	*	*	*	*	** (age, education)	* -	*	-	Good
Schwartz et al., 2018 [83]	*	*	-	*	** (age, education)	* *	*	-	Good
Srivastava et al., 2018 [84]	*	*	*	*	* (age)	* *	*	-	Good
Araga et al., 2019 [85]	*	*	-	*	-	* -	*	-	Poor
Drew et al., 2020 [55]	*	*	-	*	-	* -	*	-	Poor
Kahya et al., 2020 [86]	*	*	*	*	** (age, sex)	* -	*	-	Good
Moon et al., 2020 [87]	*	*	*	*	* (age)	* -	*	-	Good

a, *Movement disorders*; b, *Parkinsonism-related disorders*; *, point awarded, ** two points awarded, -, point not awarded.

## Data Availability

Data from the systematic search and meta-analysis are available upon justified request.

## Abbreviations

**Acronym**	**Meaning**
AHRQ	Agency for Healthcare Research and Quality
AMax	Maximum Acceleration of Constriction
CAmp	Constriction Amplitude
CBD	Corticobasal Disease
CI	Confidence Interval
CL	Constriction Latency
DaT-SPECT	Dopamine Transporter Single-Photon Emission Computed Tomography
DLB	Dementia with Lewy Bodies
DPE	Dilated Pupil Equilibrium
DPE	Dipivefrine Hydrochloride
H&Y	Hoehn and Yahr
HC	Healthy Controls
ICA	Initial Constriction Amplitude
ipRGCs	Intrinsically Photosensitive Retinal Ganglion Cells
L-DOPA	Levodopa
MMSE	Mini-Mental State Examination
MRI	Magnetic Resonance Imaging
MSA	Multiple System Atrophy
NCI	Normalised Constriction Index
PD	Parkinson’s Disease
PL	Pilocarpine Hydrochloride
PLR	Pupillary Light Reflex
PSP	Progressive Supranuclear Palsy
REM	Rapid Eye Movements
RM	Response Mean
RMax	Maximum Response Pupil Size
SMD	Standardised Mean Difference
SMD	Standardised Mean Difference
UPDRS	Unified Parkinson’s Disease Rating Scale
VMax	Maximum Constriction Velocity
